# Combined use of eDNA metabarcoding and video surveillance for the assessment of fish biodiversity

**DOI:** 10.1111/cobi.13183

**Published:** 2018-09-12

**Authors:** Michael Stat, Jeffrey John, Joseph D. DiBattista, Stephen J. Newman, Michael Bunce, Euan S. Harvey

**Affiliations:** ^1^ Trace and Environmental DNA (TrEnD) Laboratory School of Molecular and Life Sciences Curtin University Perth Western Australia 6102 Australia; ^2^ Department of Biological Sciences Macquarie University North Ryde New South Wales 2109 Australia; ^3^ Australian Museum Research Institute Australian Museum Sydney New South Wales 2010 Australia; ^4^ Western Australian Fisheries and Marine Research Laboratories Department of Primary Industries and Regional Development Government of Western Australia P.O. Box 20 North Beach Western Australia 6920 Australia

**Keywords:** baited remote underwater video systems, elasmobranchs, environmental DNA, environmental genomics, marine management, ADN ambiental, elasmobranquios, genómica ambiental, manejo marino, sistemas remotos de video submarino con carnada

## Abstract

Monitoring communities of fish is important for the management and sustainability of fisheries and marine ecosystems. Baited remote underwater video systems (BRUVs) are among the most effective nondestructive techniques for sampling bony fishes and elasmobranchs (sharks, rays, and skates). However, BRUVs sample visually conspicuous biota; hence, some taxa are undersampled or not recorded at all. We compared the diversity of fishes characterized using BRUVs with diversity detected via environmental DNA (eDNA) metabarcoding. We sampled seawater and captured BRUVs imagery at 48 locales that included reef and seagrass beds inside and outside a marine reserve (Jurien Bay in Western Australia). Eighty‐two fish genera from 13 orders were detected, and the community of fishes described using eDNA and BRUVs combined yielded >30% more generic richness than when either method was used alone. Rather than detecting a homogenous genetic signature, the eDNA assemblages mirrored the BRUVs’ spatial explicitness; differentiation of taxa between seagrass and reef was clear despite the relatively small geographical scale of the study site (∼35 km^2^). Taxa that were not sampled by one approach, due to limitations and biases intrinsic to the method, were often detected with the other. Therefore, using BRUVs and eDNA in concert provides a more holistic view of vertebrate marine communities across habitats. Both methods are noninvasive, which enhances their potential for widespread implementation in the surveillance of marine ecosystems.

## Abstract 2

*Article impact statement*: Use of video and environmental genomics in conservation will greatly improve capacity to effectively monitor fish biodiversity.

## Abstract (es)

Uso Combinado del Metacódigo de Barras de eDNA y Videograbaciones para la Evaluación de la Biodiversidad de Peces

**Resumen**

El monitoreo de comunidades de peces es importante para el manejo y sustentabilidad de las pesquerías y los ecosistemas marinos. Los sistemas remotos de video submarino con carnada (SRVSC) están entre las técnicas no destructivas más efectivas para el muestreo de peces óseos y elasmobranquios (tiburones, mantarrayas y rayas). Sin embargo, los SRVSC muestrean biota que es conspicua visiblemente; entonces, algunos taxones están mal muestreados o simplemente no se registran en los muestreos. Comparamos la diversidad de peces caracterizada usando SRVSC con la diversidad detectada por medio del metacódigo de barras de ADN ambiental (eDNA, en inglés). Muestreamos el agua de mar y capturamos imágenes con SRVSC en 48 localidades que incluyeron el arrecife y los pastos marinos dentro y fuera de una reserva marina (Bahía Jurien en el oeste de Australia). Se detectaron 83 géneros de peces de 13 órdenes, y la comunidad de peces descrita con el uso combinado del eDNA y el SRVSC produjo >30% riqueza más genérica que cuando cualquiera de los dos métodos se usó individualmente. En lugar de detectar una firma genética homogénea, los ensamblados de eDNA reflejaron la claridad espacial del SRVSC; la diferenciación de los taxones entre los pastos marinos y el arrecife fue clara a pesar la escala geográfica relativamente pequeña del sitio de estudio (∼35 km^2^). Los taxones que no fueron muestreados por uno de los métodos, por causa de limitaciones y sesgos intrínsecos al método, casi siempre fueron detectados usando el otro método. Por lo tanto, el uso de SRVSC y el eDNA en concreto proporciona una visión más holística de las comunidades marinas de vertebrados en todos los hábitats. Ambos métodos son no invasivos, lo que incrementa su potencial para ser una implementación de uso amplio en la vigilancia de los ecosistemas marinos.

## Introduction

Effective management depends on information derived from an ecosystem, including its community structure and interactions between biotic and abiotic factors (Carr 2000). Biodiversity indicators are commonly used as proxies to assess the condition of an ecosystem due to the challenges of performing whole‐ecosystem audits (Henle et al. 2013; Mallet & Pelletier 2014). Fish have recreational, commercial, and cultural significance and are globally important for food security (Garcia & Rosenberg 2010). Fish assemblages span a range of trophic levels from planktivores and herbivores to top‐order predators and are sensitive to human disturbance (Harris 1995). Fish monitoring can be extractive or observational (Murphy & Jenkins 2010; Mallet & Pelletier 2014). A nondestructive sampling technique that is now widely implemented to survey and monitor bony fishes and elasmobranchs (i.e., sharks, skates, and rays) is baited remote underwater video systems (BRUVs) (Murphy & Jenkins 2010; Warnock et al. 2016). When used in combination with stereo video (Harvey & Shortis 1995), BRUVs record the relative abundance of a broad range of fishes that inhabit different habitats and depths (Watson et al. 2005; Harvey et al. 2007) and estimate lengths and relative biomass of fishes (Bornt et al. 2015; Langlois et al. 2015). For example, global initiatives, such as Global FinPrint (www.globalfinprint.org), use BRUVs as the preferred method to collect data in a standardized manner. However, BRUVs have a known set of biases, and even though some visually conspicuous fishes are recorded, smaller, more cryptic species are often missed (Harvey et al. 2007; Stobart et al. 2007). To effectively monitor marine vertebrates, there is a need for techniques that are nondestructive, inexpensive, and rapid that generates accurate, unbiased, and high‐resolution data (Murphy & Jenkins 2010; Dickens et al. 2011).

The analysis of environmental DNA (eDNA) may help resolve a wide range of ecological questions relating to biodiversity, diet, the detection of invasive species, and population genetics (e.g., Jerde et al. 2011; Bohmann et al. 2014). Such analyses are feasible for eukaryotes because they leave detectable levels of DNA in the environment (Ficetola et al. 2008; Taberlet et al. 2012). When eDNA is combined with metabarcoding, the entire biodiversity of an ecosystem can be examined (e.g., Drummond et al. 2015; Stat et al. 2017). Thus, metabarcoding eDNA from the marine environment is a promising avenue for monitoring the ocean's biodiversity (Valentini et al. 2016).

A number of studies have demonstrated the utility of using eDNA metabarcoding to assess fish diversity (e.g., Thomsen et al. 2012; Miya et al. 2015; Evans et al. 2016). In some cases, eDNA analysis is superior to traditional techniques for characterizing fish diversity (e.g., trawling, line fishing, and diver observations) (Thomsen et al. 2012; Shaw et al. 2016). In other cases, eDNA results were comparable to the diversity of fishes caught via trawling (Thomsen et al. 2016) and visual dive surveys (Port et al. 2016). Although Kelly et al. (2017) found eDNA metabarcoding recovers a higher diversity of taxa than manual tow nets, the two methods collectively yielded more information on the biota present, suggesting a combined approach that includes eDNA provides a more holistic view of marine communities.

**Figure 1:**
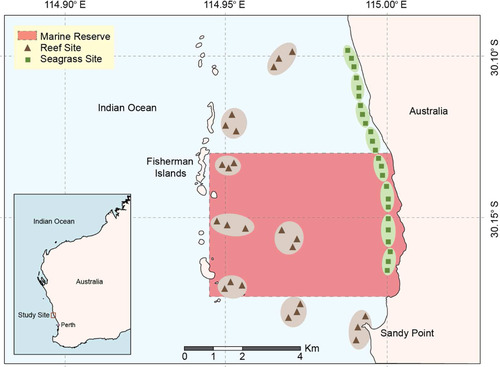
Location of seawater and baited remote underwater video system samples collected in Jurien Bay, Western Australia (48 locales; 24 in reefs and 24 in seagrass beds). Locales are grouped into 16 sites (8 seagrass and 8 reef inside and outside the marine reserve).

We compared the diversity of marine fishes detected using stereo‐BRUVs with eDNA concurrently collected from seawater and characterized using metabarcoding. Our study area was Jurien Bay Marine Park (JBMP) in Western Australia (Fig. 1), which encompasses a variety of marine habitats, hosts economically important temperate kelp forests (Bennett et al. 2015) and seagrass meadows (Hyndes et al. 2016), and contains marine reserves. Specifically, the fish taxa detected using BRUVs and eDNA metabarcoding were compared to determine the ability of the methods to differentiate fish assemblages between adjacent reefs and seagrass beds.

## Methods

### Field Site and Sample Collection

Seawater for the analysis of eDNA was collected first followed by the deployment of BRUVs from each locale within JBMP. We collected 500 mL of surface water in a sterile bottle and stored it on ice before deploying BRUVs. At the end of each day (<8 h), seawater samples were filtered across a 0.45‐μm Supor hydrophilic polyethersulfone membrane (Pall, New York) with a Sentino peristaltic pump (Pall), and the filter paper was stored at –20 °C until genomic DNA extraction. For stereo‐BRUVs, Sony CX 12 high‐definition video cameras in purpose built underwater housings separated by 0.7 m and orientated at an 8° inward angle were deployed as in Bornt et al. (2015). Approximately 1 kg of crushed pilchards (*Sardinops sagax*) was placed in a bait bag and suspended on a steel arm 1.2 m in front of the cameras (Hardinge et al. 2013). The stereo‐BRUVs were deployed by a rope and left on the seafloor to record the field of view for 1 h.

Paired eDNA and stereo‐BRUVs samples were collected from 48 locales at least 250 m apart to minimize fish being attracted by one camera and then another (Harvey et al. 2007). Locales included 24 rocky reefs dominated by macroalgae and 24 seagrass beds. Locales were grouped into 8 reef and 8 seagrass sites (3 locales in each site) evenly distributed inside and outside the marine reserve. All samples were collected over 2 d (23–24 May 2015).

### Image Analysis

All footage from the left and right cameras on stereo‐BRUVs were converted from AVCHD (.mts) format to Xvid (.avi) format with Xilisoft Video Converter Ultimate (www.xilisoft.com) and analyzed using EventMeasure (Stereo) software (SeaGIS, Bacchus Marsh, Australia). Fish were identified to the lowest taxonomic classification possible.

### DNA Extraction and Metabarcoding

We extracted DNA from seawater with a DNeasy Blood and Tissue Kit (Qiagen, Venlo, Netherlands). Half the filter was immersed in 2× lysis buffer and proteinase K, incubated at 56 °C for 3 h, washed, and eluted in 100 μL of AE buffer. Blank controls (i.e., no sample) were coextracted alongside the samples.

Metabarcoding was performed in duplicate on each DNA extract with the fish‐specific primers 16SF/D (5′ GAC CCT ATG GAG CTT TAG AC 3′) (Berry et al. 2017) and 16S2R‐degenerate (5′ CGC TGT TAT CCC TAD RGT AAC T 3′) (Deagle et al. 2007) that target the 16S rDNA region of the mitochondrial genome. Polymerase chain reaction (PCR) was performed on a StepOnePlus Real‐Time PCR System (Applied Biosystems, Waltham, Massachusetts) with fusion tag primers consisting of Illumina adaptors, indexes unique to this study, and the fish‐specific primers. The PCR conditions and reagents used were as in Stat et al. (2017). Negative and extraction controls included showed no evidence of amplification.

Fish 16S amplicons were pooled in equal concentration, size‐selected using a Pippin Prep (Sage Science, Beverly, Massachusetts), and purified using the Qiaquick PCR Purification Kit (Qiagen). The final library was sequenced unidirectionally with a 300 cycle MiSeq V2 Reagent Kit and nano flow cell on an Illumina MiSeq platform. Raw sequence data are available from https://doi.org/10.5061/dryad.c95g653.

### Bioinformatics

Sequences containing 100% identity to the fusion tag primers identified using Geneious 8.1.4. (Kearse et al. 2012) were kept for downstream analyses. Mothur 1.36.1 (Schloss et al. 2009) was used to remove singletons, sequences with an average *Q* score ≤25, reads with ambiguous bases, and chimeras identified using Perseus (Quince et al. 2011). The remaining sequences were queried against the National Center for Biotechnology Information nucleotide database with the blastn tool. To improve the availability of reference barcodes, DNA from the tissue of 302 fish species collected in Western Australia were extracted, amplified, and sequenced as above, and a reference haplotype for each species determined as in Stat et al. (2017). Fish 16S amplicons from JBMP were also queried against the west Australian custom fish database with blastn. Sequences were assigned to a species if there was ≥99% sequence identity across the entire length of the amplicon to a reference barcode, if a sequence from at least one other species within the same genus was available for comparison (and <99% identical), and if the distribution of the species matched online database records for Jurien Bay (Atlas of Living Australia [http://www.ala.org.au/]). If a species assignment could not be recorded, the taxonomic resolution was collapsed to the genus level if the amplicon maintained a ≥95% match across its entire length to a reference barcode. All sequences that assigned to the *Sardinops* genus, despite being found in JBMP, were removed from the data set because we could not rule out contamination from bait.

### Statistical Analyses

The presence or absence of fish genera based on BRUVs and eDNA was determined for each locale. Univariate analyses were performed using R (R Development Core Team 2008) and the vegan community ecology package (Oksanen et al. 2007). Presence–absence data were also used to generate a Bray–Curtis similarity matrix in PRIMER version 6 (Clarke & Gorley 2015). A permutational multivariate analysis of variance (PERMANOVA) with factors method (eDNA vs. BRUV), habitat (reef vs. seagrass), protection (inside vs. outside the marine reserve), and their interactions was performed using the PERMANOVA+ add on in PRIMER (Anderson et al. 2008). All PERMANOVA tests were conducted using unrestricted permutation of raw data and 9999 permutations. We conducted canonical analysis of principle coordinates, leave‐one‐out and SIMPER analyses. We also calculated the significance of the trace statistic (tr[Q_m'HQ_m]), a measure of the differences among groups in multivariate space (Anderson et al. 2008), with PERMANOVA+.

## Results

### Fish Diversity

**Figure 2:**
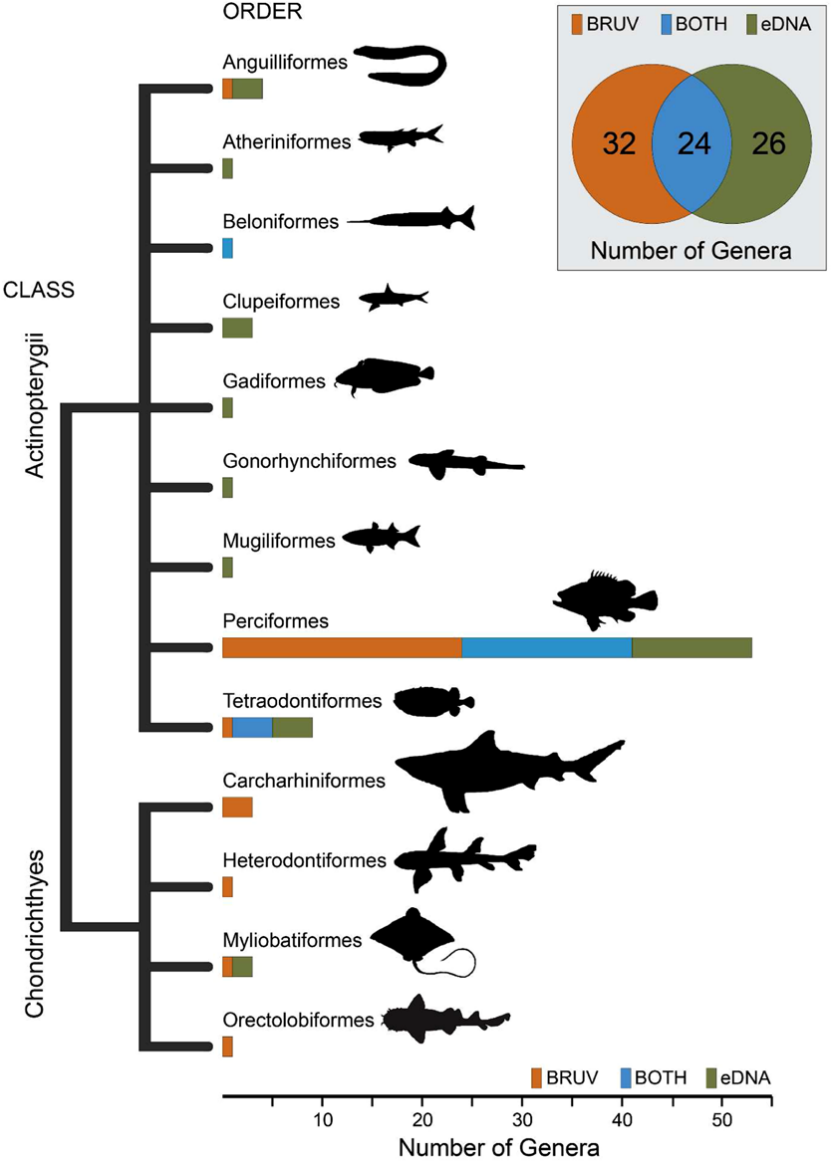
Dendrogram of fish diversity detected from samples collected using baited remote underwater video systems (BRUVs), eDNA, and both methods at Jurien Bay in Western Australia. The inset shows the number of genera identified using BRUVs, eDNA, and both methods in combination.

The eDNA metabarcoding yielded 740,847 16S rDNA sequences from 41 samples; 3 reef and 4 seagrass samples failed to amplify. The mean (SD) number of sequences per sample was 16,893 (5819) based on 692,648 reads that passed quality filtering. For BRUVs, 4974 fish were observed across all samples, and 73 taxa were identified to genera or species level, whereas 55 taxa were identified using eDNA (Supporting Information). Because only ∼40% of the fish taxa could be resolved to species level with eDNA (∼96% for BRUVs), all analyses were performed at the genus level to facilitate comparisons between methods. When combining BRUV and eDNA data sets, 82 fish genera from 13 orders and 2 classes (Actinopterygii and Chondrichthyes) were recorded (Fig. 2). Twenty‐four (29.3%) fish genera resolved with both eDNA and BRUVs, and an additional 32 (39.0%) and 26 (31.7%) genera were identified with only BRUVs and eDNA, respectively. The number of fish genera detected per sample with BRUVs (mean = 11.67 [4.17]) was significantly higher than that with eDNA (mean = 4.5 [3.26]; Mann–Whitney–Wilcoxon Test, *W* = 187.5, *p* < 0.05).

**Figure 3:**
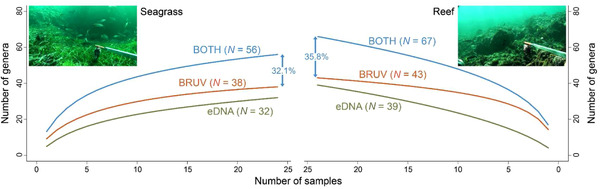
Species accumulation curves for the number of fish genera detected at Jurien Bay in Western Australia with eDNA and baited remote underwater video systems (BRUVs) in seagrass beds and reefs (numbers in parentheses indicate total number of genera detected using eDNA, BRUVs, or both methods combined; percentages indicate the increase in taxa identified between BRUVs and both methods combined).

The number of fish genera identified using BRUVs in seagrass (*n* = 38) and reef (*n* = 43) was greater than with eDNA (*n* = 32, *n* = 39, respectively), and significantly more taxa were detected per sample with BRUVs than with eDNA for reef (Mann–Whitney–Wilcoxon Test, *W* = 562, *p* < 0.05) and seagrass (Mann–Whitney–Wilcoxon Test, *W* = 491, *p* < 0.05). A significant difference between reef (mean = 14.21 [3.90]) and seagrass (mean = 9.125 [2.64]) in the number of fish genera per sample was detected with BRUVs (2‐sample *t*‐test, *t* = 5.2855, *p* < 0.05) but not with eDNA (reef = mean 4.08 [3.08]; seagrass mean = 4.92 [3.44]). The species accumulation curves for the number of fish genera detected for each method and habitat appeared close to saturation with the exception of eDNA across reef locales (Fig. 3). Although BRUVs captured a higher diversity of fish, the taxa identified using eDNA added a further 18 and 24 fish genera recorded in JBMP for seagrass and reef, respectively (32.1% and 35.8% increase in recovered taxa, respectively) (Fig. 3).

### Fish Communities

**Figure 4:**
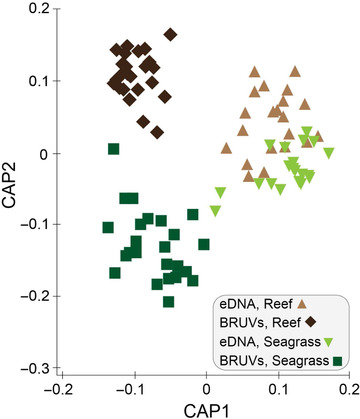
Results of canonical analysis of principle coordinates ordination plot showing the relationship of fish (genera) assemblages identified in each sample from Jurien Bay in Western Australia based on a Bray–Curtis similarity matrix for factors method (eDNA vs. BRUVs) and habitat (seagrass vs. reef).

The composition of fish genera was significantly different between eDNA and BRUV samples (*p* < 0.005) between reef and seagrass (*p* < 0.005) and for the interaction between method (eDNA vs. BRUVs) and habitat (*p* < 0.005) (Table 1). All pairwise tests for the term method × habitat were significant (*p* < 0.005) (Supporting Information). There was a clear separation in ordination between the samples detected using eDNA and BRUVs and for habitat (Fig. 4). Even though there was some overlap in the communities of fish detected using eDNA between reef and seagrass, indicating less habitat resolving power than BRUVs, the composition was still significantly different (*p* < 0.005) (Supporting Information). The separation of samples by group (Fig. 4) was reflected in the leave‐one‐out allocation results, where 80.95% of eDNA reef samples, 90.00% of eDNA seagrass samples, 95.85% of BRUVs seagrass samples, and 100.00% of BRUVs reef samples were correctly assigned; overall correct sample allocation was 92.14%. Finally, differences among the 4 groups in multivariate space were significant (permutation test trace statistic tr[Q_m'HQ_m] = 2.32554, *p* < 0.005).

**Table 1:** PERMANOVA results of fish assemblages at Jurien Bay in Western Australia for the factors method (eDNA vs. BRUV), habitat (reef vs. seagrass), protection (inside vs. outside of the marine reserve), and their interactions

Source	df	Pseudo‐*F*	P (perm)^a^
Method	1	15.686	0.0001^b^
Habitat	1	11.837	0.0003^b^
Protection	1	0.70236	0.5867
Method × habitat	1	7.2988	0.0001^b^
Method × protection	1	1.2276	0.2759
Habitat × protection	1	0.77679	0.5318
Site (habitat × protection)	12	1.5661	0.0002^b^
Method × habitat × protection	1	0.78766	0.5987
Method × site (habitat × protection)	12	1.5047	0.0028^b^

a Permutation *p* value.

b Significant factors and interactions (*p* < 0.005).

*Coris* and *Torguigener* were identified as genera that contributed the greatest dissimilarity (5.09% and 4.85%, respectively) between the eDNA and BRUVs fish assemblages, even though both were detected using each method (Supporting Information). The occurrence of *Atherinomorus* and *Parma* contributed the highest average abundance in seagrass and reef (SIMPER analysis; 19.10% and 31.81%, respectively) for fish assemblages characterized using eDNA and contributed the greatest dissimilarity between these 2 groups (SIMPER analysis; 6.66% and 6.41%, respectively). For BRUVs, whereas *Torguigener* and *Coris* contributed the highest average fish abundance in seagrass and reef (SIMPER analysis; 22.52% and 12.65%, respectively), *Scobinichthys* and *Epinephelides* accounted for the greatest dissimilarity between habitats (5.04% and 4.99%, respectively). Finally, for shared taxa identified with both eDNA and BRUVs, there was no relationship observed in the frequency at which each fish genus was detected between methods across seagrass or reef locales (Supporting Information). Specifically, 79% and 69% of the shared taxa were more consistently detected with BRUVs than with eDNA for both seagrass and reef, respectively.

## Discussion

Fish surveys globally are increasingly relying on nondestructive observational techniques to characterize and monitor communities in order to inform on the health of fisheries and marine ecosystems. Here, we show that a more holistic view of fish assemblages is achievable when BRUVs and eDNA are used in tandem, and that each method is capable of resolving distinct communities over small spatial scales (1–10 km) that are related to habitat type.

### Complementarity of eDNA and BRUVs Fish Surveys

We found surveys of fish that were characterized using eDNA and BRUVs to be highly complementary, rather than labeling a single approach as superior. It is likely that biases and limitations of each methodology select for different fishes. For example, BRUVs record fish that are mobile, visually conspicuous, whose home ranges overlap with the field of view of the cameras, as well as species attracted to the bait or the activity of other fish around the bait bag (Harvey et al. 2007; Hardinge et al. 2013; Cundy et al. 2017). For eDNA, PCR amplification biases due to primer choice as well as the low copy number of eDNA templates leads to preferential and stochastic amplification of DNA from some fish over others. For example, 3 orders of fish within the Chondrichthyes were only identified with BRUVs. As in other studies (e.g., Kelly et al. 2014*a*; Miya et al. 2015), detecting Chondrichthyes is challenging with primers that are designed for the Actinopterygii. In this study, the forward primer employed has either 1 or 2 mismatches to DNA from taxa within the Chondrichthyes, and therefore preferential binding and amplification of Actinopterygii will occur during PCR. Similarly, in a study by Kelly et al. (2014*a*), all 3 of the Carcharhiniformes present in the Monterey Bay Aquarium were undetected, whereas 7 of the 8 Actinopterygii were detected. Consistent with our findings, Kelly et al. (2014*a*) suggested that mismatches in the primer binding region of Carcharhiniformes DNA with the vertebrate 12S primers used lead to preferential amplification of Actinopterygii. Recognizing the challenges of using a single primer set for all fishes, Miya et al. (2015) developed several 12S assays and used them in combination to assess fish diversity. Therefore, to increase the likelihood of characterizing a greater diversity of fish, that is inclusive of both bony fishes and elasmobranchs, multiple metabarcoding assays should be utilized coupled with in silico assessment of potential primer binding bias (Miya et al. 2015; Shaw et al. 2016; Stat et al. 2017). However, the DNA yield of different fishes can also impact detection rates as the same PCR assay used in this study that did not recover many of the Carcharhiniformes as well as some Actinopterygii (e.g., *Chlorurus*, *Lethrinus*, and *Abudefduf*) did in fact detect these in a previous study (Stat et al. 2017). Another reason that may account for missed taxa using eDNA when compared with BRUVs is the behavior of fish. Because eDNA detections are sensitive to the copy number and dispersal of DNA in the water column, predatory or pelagic fish, such as *Grammatorcynus bicarinatus*, *Thunnus tonggol*, and *Negaprion acutidens*, and species in the genus *Epinephelus* and *Carcharhinus* that are likely to be rare on inshore rocky reefs, may not have remained long enough in the area where water was collected to leave detectable levels of DNA. In contrast, when BRUVs were deployed following the collection of seawater, these fish would have been attracted to the bait and thus recorded. Hence, the residency time and movement patterns of some fish species likely explain some of the differences seen in the fish taxa detected between methods.

Notable fish that were detected with eDNA include small, cryptic, and nocturnal taxa, such as *Atherinmorus pinguis*, *Etrumeus teres*, and *Priacanthus sagittarius*, which are difficult to observe and usually not recorded using BRUVs (Watson et al. 2005; Harvey et al. 2007). Similar to the results reported here, other studies that have surveyed fish communities using eDNA have proven that cryptic fish fauna are readily detected using genetic methods (e.g., Port et al. 2016; DiBattista et al. 2017; Yamamoto et al. 2017). Taken together, our study demonstrates that the fishes identified with either BRUVs or eDNA overcome some of the limitations in detecting them with the other method, and hence when employed together provide a more holistic view of the fish taxa that are present in an ecosystem.

The lack of repeatable detections of specific fish taxa with eDNA in this study may be due to a number of factors, including differential shedding and defecation rates (Sassoubre et al. 2016) and the volume of water that was filtered (DiBattista et al. 2017). A low yield of fish DNA (based on qPCR, data not shown) and the relatively low number of fish that were recorded per sample likely accounts for the stochasticity in fishes that were detected with eDNA and the overall lack of correlation between the frequency of fish genera between methods (Peccoud & Jacob 1996; Taberlet et al. 1996; Deagle et al. 2006). Therefore, filtering larger volumes of water and performing additional replicates should be implemented in future studies as this will result in more reliable detections of fish using eDNA (Goldberg et al. 2016; Port et al. 2016; Alberdi et al. 2017). However, it is difficult to predict a priori what volume of seawater and number of replicates is needed to generate a robust eDNA data set for any given habitat especially given the modest number of open water eDNA studies on fishes conducted across small spatial scales.

### Spatial Structure and Habitat Partitioning of Fish Communities

We found that BRUVs and eDNA were able to differentiate fish communities associated with reef or seagrass, demonstrating the capacity of both techniques to be ecologically informative, even over small spatial scales (∼35 km^2^). For example, both eDNA and BRUVs recovered fish in the genus *Parma* from reef locales only, which is consistent with previous findings (Saunders et al. 2014). To highlight the complementarity of the techniques, taxa driving habitat differences were only found with 1 method; *Atherinomorus pinguis* was identified only from seagrass with eDNA and *Epinephelides* was identified only from reef with BRUVs. Although some researchers report that protected marine areas harbor a higher diversity of fish compared with unprotected areas (Watson et al. 2009; Bornt et al. 2015), we did not find a difference across protection status in JBMP with either eDNA or BRUVs, which is also consistent with other studies (Edgar & Barrett 2012; Cundy et al. 2017). This may be due to a range of factors, such as levels of extractive fishing in the area, the small size of the marine reserve, or migration out of the protected areas (Edgar & Barrett 2012; McLaren et al. 2015).

Our study contributes to the growing evidence of spatial organization of eDNA in the ocean, rather than there being a homogenous pool as a result of water movement and mixing (i.e., tides, currents, and upwelling). Consistent with our study, Port et al. (2016) found differences in the community of vertebrates (birds, mammals, and fish) associated with seagrass, reef, and kelp. Furthermore, spatial differences in the biotic composition characterized using eDNA have been shown to occur across depths, and decrease in similarity with distance (Andruszkiewicz et al. 2017, O'Donnell et al. 2017). It is unclear what the primary drivers are for the spatially explicit fish eDNA data. We suggest that DNA is degrading rapidly in the ocean and provides an accurate but short‐lived representation of spatially distinct species assemblages or that the low copy number of fish mtDNA in the water column may mean dilution effects result in a rapid drop in detection away from the source until the sensitivity thresholds of the metabarcoding assay are reached. In addition, particles suspended in the water column (the most likely source of eDNA) may not be overly mobile and not readily homogenized. Additional research and further empirical data are required to determine the dynamics and distribution of eDNA in marine environments.

Nondestructive methods that can accurately record and measure biodiversity in the ocean represent the future of marine biomonitoring. Our data demonstrate that the analysis of eDNA through metabarcoding is a powerful complementary tool to BRUVs for assessing fish communities. The >30% increase in identified fish taxa when eDNA data were added to BRUV data clearly demonstrated the capacity to better resolve fish communities when both methods are employed. Depending on the question at hand, adding eDNA data sets in the form of species richness, which range in the sum of AU $40–100 per sample (influenced by library preparation method, sequencing depth, and the number of PCR assays), has much to offer fisheries management. This is a marginal additional cost compared with the AU$200 (low diversity sites) to AU$500 (high diversity sites) to collect and analyze the imagery from a single BRUV deployment. The combined approach should be considered for programs that survey fish communities over spatial and temporal scales, investigate the impact and disturbance to marine habitats from anthropogenic and climatic factors, and, through the diversity and specific taxa of fish present, identify areas of high species richness and assess the health of marine ecosystems. However, methodological development of both approaches will further advance the speed and resolution at which fishes can be detected. Metabarcoding of eDNA is an emerging tool in fisheries management and still needs to overcome a number of hurdles surrounding assay design, sensitivity, eDNA movement, sampling strategies, taxon assignments, DNA turnover, and optimal workflows (Kelly et al. 2014*b*; Barnes & Turner 2016; Goldberg et al. 2016). For BRUVs, a major step forward would involve increasing the speed at which fish detections were recorded through the use of computer learning algorithms that automatically score and record species and their biomass (Salman et al. 2016; Siddiqui et al. 2017). However, the power of using these 2 approaches would only be realized through the codevelopment of technologies to both record movies and capture molecules—a stereo‐BRUV system that incorporates a filtration apparatus for the collection of eDNA. Finally, eDNA, unlike BRUV's, can be used to assess taxa other than fish. Although not the focus of this study, the ability of eDNA metabarcoding to describe biota across the tree of life (i.e., ToL‐metabarcoding [Stat et al. 2017]) means that the approach as a proxy for measuring biodiversity has a lot to offer marine surveillance.

## Supporting information

Fish taxa (Appendix S1), PERMANOVA pairwise tests (Appendix S2), frequency (Appendix S3), relationship of fish detections using eDNA and BRUVs (Appendix S4), and the Simper analysis output (Appendix S5) are available online. The authors are solely responsible for the content and functionality of these materials. Queries (other than absence of the material) should be directed to the corresponding author.
