# Supplementary material for: Combined use of eDNA metabarcoding and video surveillance for the assessment of fish biodiversity
Source: Conserv Biol. 2018 Sep 12;33(1):196–205. doi: 10.1111/cobi.13183 (PMC7379492; doi:10.1111/cobi.13183)
Supplement: Supplementary file 1 — Fish taxa (Appendix S1), PERMANOVA pairwise tests (Appendix S2), frequency (Appendix S3), relationship of fish detections using eDNA and BRUVs (Appendix S4), and the Simper analysis output (Appendix S5) are available online. The authors are solely responsible for the content and functionality of these materials. Queries (other than absence of the material) should be directed to the corresponding author. [file COBI-33-196-s001.pdf]

Appendix S1. Fish taxa identified in the samples collected in this study at Jurien Bay in west Australia. Species names represent nomenclature as extracted from NCBI Genbank.

Class	Order	Family	Genus	Species	eDNA	BRUV
Actinopterygii	Anguilliformes	Congridae	*Conger*	*japonicus*	X	
Actinopterygii	Anguilliformes	Congridae	*Gnathophis*		X	
Actinopterygii	Anguilliformes	Muraenidae	*Gymnothorax*	*prasinus*		X
Actinopterygii	Anguilliformes	Muraenidae	*Gymnothorax*	*undulatus*		X
Actinopterygii	Anguilliformes	Muraenidae	*Gymnothorax*	*woodwardi*		X
Actinopterygii	Anguilliformes	Ophichthidae	*Scolecenchelys*		X	
Actinopterygii	Atheriniformes	Atherinidae	*Atherinomorus*	*pinguis*	X	
Actinopterygii	Beloniformes	Hemiramphidae	*Hyporhamphus*	*melanochir*	X	X
Actinopterygii	Clupeiformes	Clupeidae	*Etrumeus*	*teres*	X	
Actinopterygii	Clupeiformes	Clupeidae	*Spratelloides*	*robustus*	X	
Actinopterygii	Clupeiformes	Engraulidae	*Engraulis*		X	
Actinopterygii	Gadiformes	Moridae	*Lotella*		X	
Actinopterygii	Gonorhynchiformes	Gonorynchidae	*Gonorynchus*		X	
Actinopterygii	Mugiliformes	Mugilidae	*Mugil*	*cephalus*	X	
Actinopterygii	Perciformes	Apogonidae	*Ostorhinchus*	*rueppellii*		X
Actinopterygii	Perciformes	Apogonidae	*Ostorhinchus*	*victoriae*		X
Actinopterygii	Perciformes	Arripidae	*Arripis*	*georgianus*	X	X
Actinopterygii	Perciformes	Blenniidae	*Cirripectes*		X	
Actinopterygii	Perciformes	Carangidae	*Pseudocaranx*		X	X
Actinopterygii	Perciformes	Carangidae	*Pseudocaranx*	*dentex*	X	X
Actinopterygii	Perciformes	Carangidae	*Seriola*	*hippos*		X
Actinopterygii	Perciformes	Chaetodontidae	*Chaetodon*	*assarius*		X
Actinopterygii	Perciformes	Cheilodactylidae	*Cheilodactylus*		X	
Actinopterygii	Perciformes	Clinidae	*Heteroclinus*		X	
Actinopterygii	Perciformes	Echeneidae	*Echeneis*	*naucrates*		X
Actinopterygii	Perciformes	Haemulidae	*Plectorhinchus*	*flavomaculatus*	X	X
Actinopterygii	Perciformes	Kyphosidae	*Kyphosus*		X	
Actinopterygii	Perciformes	Kyphosidae	*Kyphosus*	*bigibbus*	X	
Actinopterygii	Perciformes	Kyphosidae	*Kyphosus*	*cornelii*	X	X
Actinopterygii	Perciformes	Kyphosidae	*Kyphosus*	*sydneyanus*		X
Actinopterygii	Perciformes	Labridae	*Anampses*	*geographicus*		X
Actinopterygii	Perciformes	Labridae	*Chlorurus*	*sordidus*		X
Actinopterygii	Perciformes	Labridae	*Choerodon*		X	
Actinopterygii	Perciformes	Labridae	*Choerodon*	*rubescens*		X
Actinopterygii	Perciformes	Labridae	*Coris*		X	
Actinopterygii	Perciformes	Labridae	*Coris*	*auricularis*		X
Actinopterygii	Perciformes	Labridae	*Haletta*		X	
Actinopterygii	Perciformes	Labridae	*Haletta*	*semifasciata*		X
Actinopterygii	Perciformes	Labridae	*Halichoeres*	*brownfieldi*	X	X
Actinopterygii	Perciformes	Labridae	*Notolabrus*		X	
Actinopterygii	Perciformes	Labridae	*Notolabrus*	*parilus*		X
Actinopterygii	Perciformes	Labridae	*Odax*		X	
Actinopterygii	Perciformes	Labridae	*Pictilabrus*		X	
Actinopterygii	Perciformes	Labridae	*Pseudolabrus*	*biserialis*		X
Actinopterygii	Perciformes	Labridae	*Thalassoma*		X	
Actinopterygii	Perciformes	Labridae	*Thalassoma*	*amblycephalum*		X
Actinopterygii	Perciformes	Labridae	*Thalassoma*	*lunare*		X
Actinopterygii	Perciformes	Labridae	*Thalassoma*	*lutescens*		X
Actinopterygii	Perciformes	Labridae	*Thalassoma*	*septemfasciatum*		X
Actinopterygii	Perciformes	Latidae	*Psammoperca*		X	
Actinopterygii	Perciformes	Latidae	*Psammoperca*	*waigiensis*		X
Actinopterygii	Perciformes	Lethrinidae	*Lethrinus*			X
Actinopterygii	Perciformes	Lethrinidae	*Lethrinus*	*nebulosus*		X
Actinopterygii	Perciformes	Microcanthidae	*Neatypus*	*obliquus*		X
Actinopterygii	Perciformes	Mullidae	*Parupeneus*	*chrysopleuron*		X
Actinopterygii	Perciformes	Mullidae	*Parupeneus*	*spilurus*		X
Actinopterygii	Perciformes	Mullidae	*Upeneichthys*	*lineatus*	X	
Actinopterygii	Perciformes	Mullidae	*Upeneichthys*	*vlamingii*		X
Actinopterygii	Perciformes	Nemipteridae	*Pentapodus*	*vitta*		X
Actinopterygii	Perciformes	Notothenioidei	*Dissostichus*		X	
Actinopterygii	Perciformes	Odacidae	*Heteroscarus*	*acroptilus*		X
Actinopterygii	Perciformes	Odacidae	*Olisthops*	*cyanomelas*		X
Actinopterygii	Perciformes	Pempheridae	*Pempheris*		X	
Actinopterygii	Perciformes	Pinguipedidae	*Parapercis*		X	
Actinopterygii	Perciformes	Pomacentridae	*Abudefduf*	*bengalensis*		X
Actinopterygii	Perciformes	Pomacentridae	*Abudefduf*	*sexfasciatus*		X
Actinopterygii	Perciformes	Pomacentridae	*Parma*	*mccullochi*	X	X
Actinopterygii	Perciformes	Pomacentridae	*Parma*	*occidentalis*	X	X
Actinopterygii	Perciformes	Pomacentridae	*Pomacentrus*		X	
Actinopterygii	Perciformes	Pomacentridae	*Pomacentrus*	*coelestis*		X
Actinopterygii	Perciformes	Pomacentridae	*Pomacentrus*	*milleri*		X
Actinopterygii	Perciformes	Priacanthidae	*Priacanthus*	*sagittarius*	X	
Actinopterygii	Perciformes	Pseudochromidae	*Labracinus*	*lineatus*		X
Actinopterygii	Perciformes	Scombridae	*Grammatorcynus*	*bicarinatus*		X
Actinopterygii	Perciformes	Scombridae	*Scomber*		X	
Actinopterygii	Perciformes	Scombridae	*Scomberomorus*	*queenslandicus*	X	
Actinopterygii	Perciformes	Scombridae	*Thunnus*	*tonggol*		X
Actinopterygii	Perciformes	Scorpididae	*Scorpis*	*georgiana*		X
Actinopterygii	Perciformes	Serranidae	*Acanthistius*	*serratus*		X

Actinopterygii	Perciformes	Serranidae	*Epinephelides*	*armatus*		X
Actinopterygii	Perciformes	Serranidae	*Epinephelus*	*fasciatus*		X
Actinopterygii	Perciformes	Serranidae	*Epinephelus*	*rivulatus*		X
Actinopterygii	Perciformes	Siganidae	*Siganus*		X	
Actinopterygii	Perciformes	Siganidae	*Siganus*	*fuscescens*	X	X
Actinopterygii	Perciformes	Sparidae	*Chrysophrys*		X	
Actinopterygii	Perciformes	Sparidae	*Pagrus*		X	
Actinopterygii	Perciformes	Sparidae	*Pagrus*	*auratus*		X
Actinopterygii	Perciformes	Sparidae	*Rhabdosargus*	*sarba*		X
Actinopterygii	Perciformes	Sphyraenidae	*Sphyraena*	*novaehollandiae*		X
Actinopterygii	Perciformes	Sphyraenidae	*Sphyraena*	*obtusata*		X
Actinopterygii	Perciformes	Sphyraenidae	*Sphyraena*	*pinguis*	X	
Actinopterygii	Perciformes	Tetrapontidae	*Pelates*	*sexlineatus*		X
Actinopterygii	Perciformes	Tetrapontidae	*Pelsartia*	*humeralis*		X
Actinopterygii	Tetraodontiformes	Diodontidae	*Diodon*	*nicthemerus*	X	
Actinopterygii	Tetraodontiformes	Diodontidae	*Tragulichthys*		X	
Actinopterygii	Tetraodontiformes	Monacanthidae	*Acanthaluteres*		X	
Actinopterygii	Tetraodontiformes	Monacanthidae	*Acanthaluteres*	*vittiger*		X
Actinopterygii	Tetraodontiformes	Monacanthidae	*Chaetodermis*		X	
Actinopterygii	Tetraodontiformes	Monacanthidae	*Meuschenia*		X	
Actinopterygii	Tetraodontiformes	Monacanthidae	*Meuschenia*	*australis*		X
Actinopterygii	Tetraodontiformes	Monacanthidae	*Meuschenia*	*galii*		X
Actinopterygii	Tetraodontiformes	Monacanthidae	*Monacanthus*		X	
Actinopterygii	Tetraodontiformes	Monacanthidae	*Scobinichthys*		X	
Actinopterygii	Tetraodontiformes	Monacanthidae	*Scobinichthys*	*granulatus*		X
Actinopterygii	Tetraodontiformes	Tetraodontidae	*Lagocephalus*	*sceleratus*		X
Actinopterygii	Tetraodontiformes	Tetraodontidae	*Torquigener*		X	
Actinopterygii	Tetraodontiformes	Tetraodontidae	*Torquigener*	*pleurogramma*		X
Chondrichthyes	Carcharhiniformes	Carcharhinidae	*Carcharhinus*			X
Chondrichthyes	Carcharhiniformes	Carcharhinidae	*Negaprion*	*acutidens*		X
Chondrichthyes	Carcharhiniformes	Triakidae	*Hypogaleus*	*hyugaensis*		X
Chondrichthyes	Heterodontiformes	Heterodontidae	*Heterodontus*	*portusjacksoni*		X
Chondrichthyes	Myliobatiformes	Dasyatidae	*Dasyatis*	*brevicaudata*	X	X
Chondrichthyes	Myliobatiformes	Myliobatidae	*Myliobatis*	*australis*	X	X
Chondrichthyes	Myliobatiformes	Urolophidae	*Trygonoptera*	*ovalis*		X
Chondrichthyes	Orectolobiformes	Orectolobidae	*Orectolobus*	*maculatus*		X
Chondrichthyes	Orectolobiformes	Orectolobidae	*Orectolobus*	*ornatus*		X
				**Total**	**55 taxa**	**73 taxa**

Appendix S2

PERMANOVA pairwise test results of fish assemblages at Jurien Bay in west Australia for the interaction of Method (eDNA versus BRUV) and Habitat (reef versus seagrass). The pseudo-*t* two-tailed test (t) result and permutation *P*-value (P(perm)) are presented. Significant factors ($P < 0.005$) are indicated with an asterisk.

**Factor**	**Level**	**Groups**	**t**	**P(perm)**
Habitat	eDNA	Reef, Seagrass	2.0259	**0.0006***
	BRUV	Reef, Seagrass	4.8809	**0.0001***
Method	Reef	eDNA, BRUV	3.1282	**0.0001***
	Seagrass	eDNA, BRUV	3.676	**0.0001***

Appendix S3

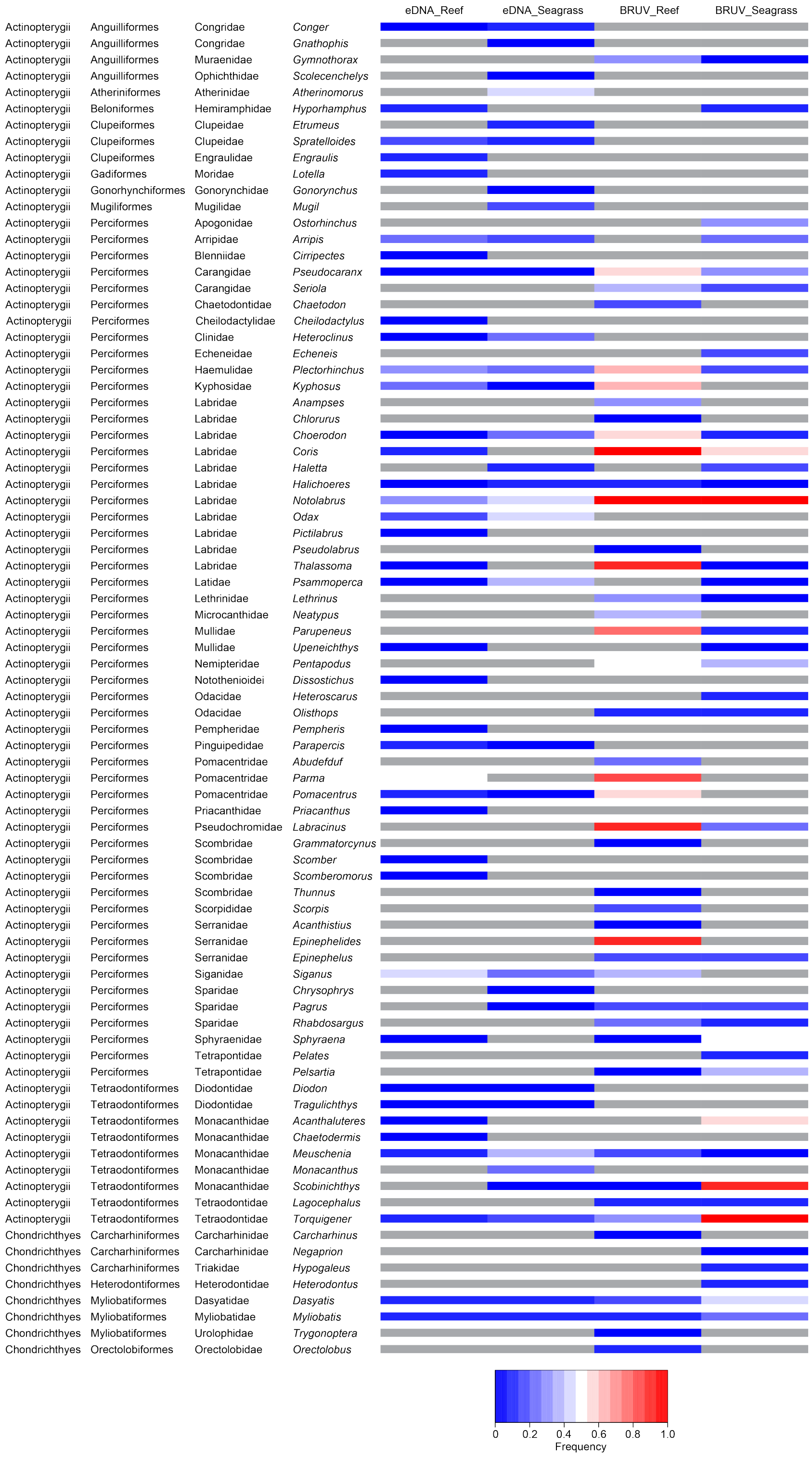

Heatmap representing the frequency of each fish genera detected in the 24 samples collected from seagrass and reef locales for both eDNA and BRUVs. Boxes shaded grey indicate no detection. The analysis was performed using the *heatmap.2* function in the package *gplots* in R (R Development Core Team 2008).

Appendix S4

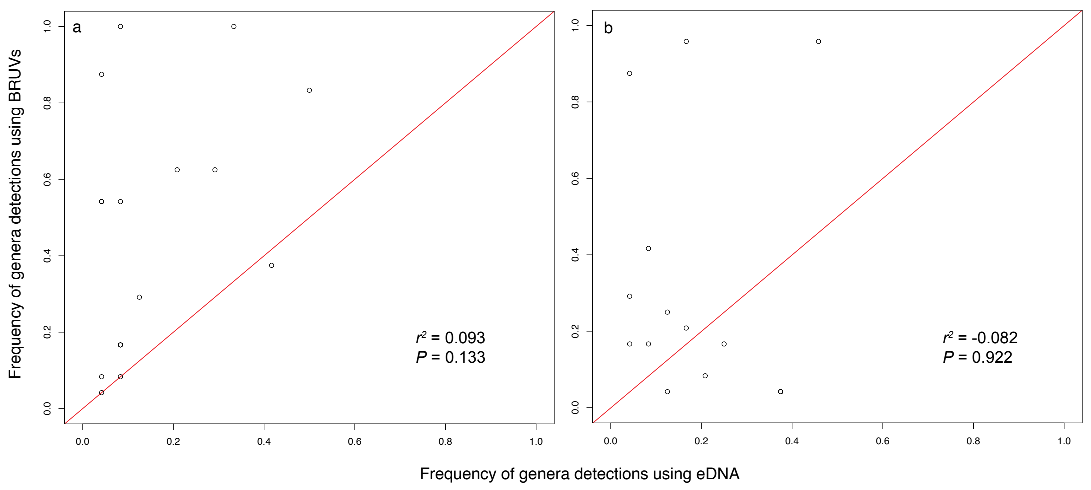

The frequency of fish genera detected using BRUVs as a function of the frequency of fish genera detected using eDNA from reef (a) and seagrass (b) locales (generated using R; R Development Core Team 2008). Only genera detected using both methods were used. Points above the red line represent fish genera detected more frequently using BRUVs and points below represent fish genera detected more frequently using eDNA.

Appendix S5 - Simper Analysis output

SIMPER - Comparison of Method (eDNA versus BRUVs)

Similarity Percentages - species contributions

One-Way Analysis

Data worksheet

Name: Jurien

Data type: Other

Sample selection: All

Variable selection: All

Parameters

Resemblance: S17 Bray Curtis similarity

Cut off for low contributions: 90.00%

Factor Groups

Sample	Method
FISPZS1R1e	eDNA
FISPZS1R4e	eDNA
FISPZS2R2e	eDNA
FISPZS2R4e	eDNA
GUZ(GH)S3R	eDNA

GUZ(GH)S3R	eDNA
GUZ(GH)S3R	eDNA
FISPZS3R1e	eDNA
FISPZS3R2e	eDNA
FISPZS3R4e	eDNA
FISZS1R1e	eDNA
FISZS1R2e	eDNA
FISZS1R3e	eDNA
GUZ(SC)S1R3	eDNA
GUZ(SC)S1R1	eDNA
GUZ(SC)S2R1	eDNA
GUZ(SC)S2R2	eDNA
GUZ(SC)S2R3	eDNA
GUZ(GH)S2R	eDNA
GUZ(GH)S2R	eDNA
GUZ(GH)S2R	eDNA
CSGS1R1e	eDNA
CSGS1R2e	eDNA
CSGS1R3e	eDNA
CSGS2R1e	eDNA
CSGS2R2e	eDNA
CSGS2R3e	eDNA
CSGS3R1e	eDNA

CSGS3R2e	eDNA
CSGS3R3e	eDNA
CSGS4R1e	eDNA
CSGS4R2e	eDNA
CSGS4R3e	eDNA
SRSGS1R3e	eDNA
SRSGS2R1e	eDNA
SRSGS2R2e	eDNA
SRSGS3R1e	eDNA
SRSGS3R3e	eDNA
SRSGS4R1e	eDNA
SRSGS4R2e	eDNA
SRSGS4R3e	eDNA
CSGS1R1b	BRUV
CSGS1R2b	BRUV
CSGS1R3b	BRUV
CSGS2R1b	BRUV
CSGS2R2b	BRUV
CSGS2R3b	BRUV
CSGS3R1b	BRUV
CSGS3R2b	BRUV
CSGS3R3b	BRUV
CSGS4R1b	BRUV

CSGS4R2b	BRUV
CSGS4R3b	BRUV
FISPZS1R1b	BRUV
FISPZS1R2b	BRUV
FISPZS1R4b	BRUV
FISPZS2R2b	BRUV
FISPZS2R3b	BRUV
FISPZS2R4b	BRUV
FISPZS3R1b	BRUV
FISPZS3R2b	BRUV
FISPZS3R4b	BRUV
FISZS1R1b	BRUV
FISZS1R2b	BRUV
FISZS1R3b	BRUV
GUZ(GH)S2R	BRUV
GUZ(GH)S2R	BRUV
GUZ(GH)S2R	BRUV
GUZ(GH)S3R	BRUV
GUZ(GH)S3R	BRUV
GUZ(GH)S3R	BRUV
GUZ(SC)S1R1	BRUV
GUZ(SC)S1R2	BRUV
GUZ(SC)S1R3	BRUV

GUZ(SC)S2R1	BRUV
GUZ(SC)S2R2	BRUV
GUZ(SC)S2R3	BRUV
SRSGS1R1b	BRUV
SRSGS1R2b	BRUV
SRSGS1R3b	BRUV
SRSGS2R1b	BRUV
SRSGS2R2b	BRUV
SRSGS2R3b	BRUV
SRSGS3R1b	BRUV
SRSGS3R2b	BRUV
SRSGS3R3b	BRUV
SRSGS4R1b	BRUV
SRSGS4R2b	BRUV
SRSGS4R3b	BRUV

Group eDNA

Average similarity: 18.20

Species	Av.Abund	Av.Sim	Sim/SD	Contrib%	Cum.%
Notolabrus	0.46	4.15	0.48	22.8	22.8
Siganus	0.39	2.44	0.39	13.41	36.21
Odax	0.34	1.88	0.34	10.3	46.51

Plectorhinchu	0.32	1.74	0.31	9.54	56.05
Parma	0.29	1.55	0.27	8.54	64.59
Atherinomor	0.27	1.18	0.26	6.46	71.05
Meuschenia	0.27	0.95	0.26	5.23	76.28
Psammoperc	0.24	0.84	0.23	4.59	80.87
Arripis	0.22	0.8	0.2	4.39	85.26
Torquigener	0.17	0.4	0.15	2.18	87.44
Kyphosus	0.15	0.28	0.13	1.54	88.98
Spratelloides	0.15	0.28	0.13	1.52	90.51

Group BRUV

Average similarity: 38.45

Species	Av.Abund	Av.Sim	Sim/SD	Contrib%	Cum.%
Notolabrus	0.98	8.59	3.01	22.35	22.35
Coris	0.77	4.75	1.15	12.36	34.71
Torquigener	0.63	4.25	0.76	11.06	45.77
Scobinichthy	0.46	2.21	0.49	5.74	51.51
Labracinus	0.54	2.16	0.62	5.62	57.13
Pentapodus	0.44	1.51	0.45	3.92	61.05
Thalassoma	0.46	1.47	0.5	3.82	64.86
Pseudocaran	0.42	1.31	0.44	3.41	68.27
Epinephelide	0.44	1.27	0.47	3.31	71.58

Parupeneus	0.42	1.2	0.44	3.12	74.7
Parma	0.42	1.2	0.44	3.11	77.81
Plectorhinchu	0.4	1.06	0.42	2.76	80.57
Acanthaluter	0.29	0.88	0.29	2.28	82.85
Dasyatis	0.29	0.85	0.29	2.21	85.06
Sphyraena	0.27	0.71	0.27	1.85	86.91
Choerodon	0.31	0.67	0.31	1.74	88.64
Kyphosus	0.31	0.6	0.32	1.56	90.2

Groups eDNA & BRUV

Average dissimilarity = 86.23

	Group eDNA	Group BRUV				
Species	Av.Abund	Av.Abund	Av.Diss	Diss/SD	Contrib%	Cum.%
Coris	0.05	0.77	4.39	1.5	5.09	5.09
Torquigener	0.17	0.63	4.18	1.07	4.85	9.94
Notolabrus	0.46	0.98	3.53	0.96	4.1	14.03
Scobinichthy	0.02	0.46	3.32	0.85	3.85	17.88
Labracinus	0	0.54	3.02	1.01	3.51	21.39
Parma	0.29	0.42	2.79	0.86	3.24	24.63
Plectorhinchu	0.32	0.4	2.77	0.86	3.21	27.84
Pentapodus	0	0.44	2.62	0.79	3.04	30.88
Siganus	0.39	0.19	2.55	0.81	2.95	33.84

Thalassoma	0.02	0.46	2.5	0.87	2.9	36.74
Pseduocaran	0.05	0.42	2.47	0.8	2.86	39.6
Dasyatis	0.1	0.29	2.29	0.66	2.65	42.26
Epinephelide	0	0.44	2.27	0.84	2.63	44.89
Parupeneus	0	0.42	2.23	0.81	2.58	47.47
Acanthaluter	0.02	0.29	2.16	0.62	2.5	49.98
Choerodon	0.15	0.31	2.06	0.72	2.39	52.36
Odax	0.34	0	2.04	0.67	2.37	54.73
Kyphosus	0.15	0.31	1.98	0.73	2.29	57.02
Sphyraena	0.02	0.27	1.96	0.59	2.27	59.29
Meuschenia	0.27	0.1	1.83	0.64	2.12	61.41
Arripis	0.22	0.1	1.76	0.58	2.04	63.45
Pomacentrus	0.07	0.27	1.64	0.62	1.9	65.35
Atherinomor	0.27	0	1.63	0.57	1.89	67.23
Myliobatis	0.12	0.17	1.58	0.53	1.84	69.07
Seriola	0	0.27	1.5	0.58	1.74	70.81
Psammoperc	0.24	0.02	1.47	0.56	1.7	72.51
Pelsartia	0	0.21	1.47	0.48	1.7	74.21
Epinephelus	0	0.17	1.04	0.43	1.2	75.41
Pagrus	0.02	0.17	0.94	0.46	1.09	76.51
Ostorhinchus	0	0.15	0.92	0.4	1.06	77.57
Neatypus	0	0.19	0.91	0.47	1.06	78.63
Lethrinus	0	0.19	0.88	0.47	1.02	79.65

Halichoeres	0.1	0.06	0.87	0.4	1.01	80.66
Spratelloides	0.15	0	0.84	0.39	0.98	81.64
Heteroclinus	0.15	0	0.82	0.4	0.95	82.59
Gymnothora:	0	0.17	0.81	0.44	0.94	83.53
Haletta	0.05	0.08	0.74	0.36	0.86	84.39
Monacanthu	0.12	0	0.72	0.36	0.84	85.23
Hyporhamph	0.07	0.04	0.72	0.33	0.83	86.06
Rhabdosargu	0	0.15	0.72	0.4	0.83	86.89
Anampses	0	0.15	0.69	0.41	0.8	87.69
Lagocephalu:	0	0.1	0.65	0.33	0.76	88.45
Abudefduf	0	0.13	0.6	0.37	0.69	89.14
Echeneis	0	0.08	0.54	0.3	0.63	89.77
Mugil	0.1	0	0.51	0.32	0.59	90.35

SIMPER - Comparison of Habitat using eDNA

Similarity Percentages - species contributions

One-Way Analysis

Data worksheet

Name: Jurien

Data type: Other

Sample selection: 1-41

Variable selection: All

Parameters

Resemblance: S17 Bray Curtis similarity

Cut off for low contributions: 90.00%

Factor Groups

Sample	Habitat
FISPZS1R1e	Reef
FISPZS1R4e	Reef
FISPZS2R2e	Reef
FISPZS2R4e	Reef
GUZ(GH)S3R[illegible]	Reef
GUZ(GH)S3R[illegible]	Reef
GUZ(GH)S3R[illegible]	Reef
FISPZS3R1e	Reef
FISPZS3R2e	Reef
FISPZS3R4e	Reef
FISZS1R1e	Reef
FISZS1R2e	Reef
FISZS1R3e	Reef
GUZ(SC)S1R3	Reef
GUZ(SC)S1R1	Reef
GUZ(SC)S2R1	Reef

GUZ(SC)S2R2	Reef
GUZ(SC)S2R3	Reef
GUZ(GH)S2R:	Reef
GUZ(GH)S2R:	Reef
GUZ(GH)S2R4	Reef
CSGS1R1e	Seagrass
CSGS1R2e	Seagrass
CSGS1R3e	Seagrass
CSGS2R1e	Seagrass
CSGS2R2e	Seagrass
CSGS2R3e	Seagrass
CSGS3R1e	Seagrass
CSGS3R2e	Seagrass
CSGS3R3e	Seagrass
CSGS4R1e	Seagrass
CSGS4R2e	Seagrass
CSGS4R3e	Seagrass
SRSGS1R3e	Seagrass
SRSGS2R1e	Seagrass
SRSGS2R2e	Seagrass
SRSGS3R1e	Seagrass
SRSGS3R3e	Seagrass
SRSGS4R1e	Seagrass

SRSGS4R2e	Seagrass
SRSGS4R3e	Seagrass

Group Reef

Average similarity: 19.07

Species	Av.Abund	Av.Sim	Sim/SD	Contrib%	Cum.%
Parma	0.57	6.07	0.6	31.81	31.81
Siganus	0.48	3.88	0.47	20.34	52.15
Notolabrus	0.38	3.3	0.37	17.3	69.45
Plectorhinchu	0.33	1.79	0.31	9.38	78.83
Arripis	0.24	1.01	0.21	5.3	84.13
Kyphosus	0.24	0.69	0.22	3.62	87.75
Odax	0.19	0.55	0.16	2.91	90.66

Group Seagrass

Average similarity: 26.57

Species	Av.Abund	Av.Sim	Sim/SD	Contrib%	Cum.%
Atherinomor	0.55	5.08	0.6	19.1	19.1
Notolabrus	0.55	4.89	0.61	18.4	37.5
Odax	0.5	3.73	0.54	14.05	51.55
Psammoperc	0.45	2.85	0.47	10.74	62.29

Meuschenia	0.45	2.58	0.47	9.72	72
Plectorhinchu	0.3	1.46	0.29	5.49	77.5
Siganus	0.3	1.15	0.29	4.32	81.81
Monacanthu	0.25	0.85	0.23	3.2	85.02
Heteroclinus	0.25	0.8	0.23	3.01	88.03
Choerodon	0.25	0.66	0.23	2.47	90.5

Groups Reef & Seagrass

Average dissimilarity = 86.02

	Group Reef	Group Seagrass				
Species	Av.Abund	Av.Abund	Av.Diss	Diss/SD	Contrib%	Cum.%
Atherinomor	0	0.55	5.73	0.98	6.66	6.66
Parma	0.57	0	5.51	0.97	6.41	13.06
Notolabrus	0.38	0.55	5.48	0.89	6.37	19.43
Odax	0.19	0.5	5.05	0.89	5.87	25.3
Siganus	0.48	0.3	4.91	0.86	5.71	31.01
Plectorhinchu	0.33	0.3	4.46	0.8	5.18	36.19
Psammoperc	0.05	0.45	4.34	0.84	5.04	41.23
Meuschenia	0.1	0.45	4.21	0.84	4.9	46.13
Arripis	0.24	0.2	3.46	0.65	4.02	50.15
Torquigener	0.14	0.2	2.99	0.5	3.47	53.62
Heteroclinus	0.05	0.25	2.54	0.57	2.95	56.57

Monacanthu	0	0.25	2.5	0.53	2.91	59.48
Choerodon	0.05	0.25	2.38	0.57	2.76	62.24
Kyphosus	0.24	0.05	2.32	0.56	2.7	64.94
Spratelloides	0.19	0.1	2.28	0.54	2.65	67.59
Myliobatis	0.1	0.15	2.18	0.49	2.54	70.13
Halichoeres	0.05	0.15	1.86	0.45	2.16	72.28
Etrumeus	0	0.15	1.77	0.39	2.06	74.34
Dasyatis	0.1	0.1	1.72	0.41	2	76.34
Mugil	0	0.2	1.62	0.47	1.88	78.22
Hyporhamph	0.14	0	1.5	0.37	1.74	79.96
Lotella	0.1	0	1.36	0.3	1.58	81.54
Conger	0.05	0.1	1.32	0.37	1.53	83.07
Pomacentrus	0.1	0.05	1.31	0.37	1.53	84.6
Coris	0.1	0	1.17	0.31	1.36	85.96
Parapercis	0.1	0.05	1.06	0.38	1.24	87.19
Engraulis	0.1	0	0.99	0.28	1.15	88.34
Pseduocaran	0.05	0.05	0.81	0.31	0.94	89.28
Haletta	0	0.1	0.71	0.31	0.82	90.1

SIMPER - Cmparison of habitat using BRUVs

Similarity Percentages - species contributions

One-Way Analysis

Data worksheet

Name: Jurien

Data type: Other

Sample selection: 42-89

Variable selection: All

Parameters

Resemblance: S17 Bray Curtis similarity

Cut off for low contributions: 90.00%

Factor Groups

Sample	Habitat
CSGS1R1b	Seagrass
CSGS1R2b	Seagrass
CSGS1R3b	Seagrass
CSGS2R1b	Seagrass
CSGS2R2b	Seagrass
CSGS2R3b	Seagrass
CSGS3R1b	Seagrass
CSGS3R2b	Seagrass
CSGS3R3b	Seagrass
CSGS4R1b	Seagrass

CSGS4R2b	Seagrass
CSGS4R3b	Seagrass
SRSGS1R1b	Seagrass
SRSGS1R2b	Seagrass
SRSGS1R3b	Seagrass
SRSGS2R1b	Seagrass
SRSGS2R2b	Seagrass
SRSGS2R3b	Seagrass
SRSGS3R1b	Seagrass
SRSGS3R2b	Seagrass
SRSGS3R3b	Seagrass
SRSGS4R1b	Seagrass
SRSGS4R2b	Seagrass
SRSGS4R3b	Seagrass
FISPZS1R1b	Reef
FISPZS1R2b	Reef
FISPZS1R4b	Reef
FISPZS2R2b	Reef
FISPZS2R3b	Reef
FISPZS2R4b	Reef
FISPZS3R1b	Reef
FISPZS3R2b	Reef
FISPZS3R4b	Reef

FISZS1R1b	Reef
FISZS1R2b	Reef
FISZS1R3b	Reef
GUZ(GH)S2R:	Reef
GUZ(GH)S2R:	Reef
GUZ(GH)S2R4	Reef
GUZ(GH)S3R:	Reef
GUZ(GH)S3R:	Reef
GUZ(GH)S3R4	Reef
GUZ(SC)S1R1	Reef
GUZ(SC)S1R2	Reef
GUZ(SC)S1R3	Reef
GUZ(SC)S2R1	Reef
GUZ(SC)S2R2	Reef
GUZ(SC)S2R3	Reef

Group Seagrass

Average similarity: 47.52

Species	Av.Abund	Av.Sim	Sim/SD	Contrib%	Cum.%
Torquigener	0.96	10.7	2.65	22.52	22.52
Notolabrus	0.96	10.15	2.75	21.35	43.88
Scobinichthy	0.88	8.53	1.63	17.95	61.83

Acanthaluter	0.58	3.59	0.68	7.55	69.38
Coris	0.54	2.71	0.62	5.71	75.09
Sphyraena	0.5	2.5	0.54	5.27	80.36
Dasyatis	0.42	1.9	0.43	4	84.36
Pentapodus	0.38	1.47	0.37	3.1	87.46
Pelsartia	0.38	1.39	0.38	2.93	90.39

Group Reef

Average similarity: 57.75

Species	Av.Abund	Av.Sim	Sim/SD	Contrib%	Cum.%
Coris	1	7.3	4.94	12.65	12.65
Notolabrus	1	7.3	4.94	12.65	25.3
Labracinus	0.88	5.4	1.65	9.35	34.64
Thalassoma	0.88	5.39	1.67	9.34	43.98
Epinephelide	0.88	5.2	1.69	9.01	52.99
Parma	0.83	4.89	1.41	8.46	61.45
Parupeneus	0.75	3.89	1.07	6.73	68.18
Plectorhinchu	0.63	2.48	0.77	4.29	72.47
Kyphosus	0.63	2.45	0.77	4.24	76.7
Choerodon	0.54	1.93	0.61	3.34	80.04
Pseduocaran	0.54	1.9	0.61	3.29	83.33
Pomacentrus	0.54	1.87	0.61	3.23	86.56

Pentapodus	0.5	1.51	0.55	2.61	89.17
Seriola	0.38	0.9	0.38	1.56	90.73

Groups Seagrass & Reef

Average dissimilarity = 75.14

	Group Seagra	Group Reef				
Species	Av.Abund	Av.Abund	Av.Diss	Diss/SD	Contrib%	Cum.%
Scobinichthy	0.88	0.04	3.78	2.05	5.04	5.04
Epinephelide	0	0.88	3.75	2.32	4.99	10.03
Thalassoma	0.04	0.88	3.72	2.06	4.95	14.97
Parma	0	0.83	3.66	1.99	4.87	19.85
Labracinus	0.21	0.88	3.23	1.47	4.29	24.14
Parupeneus	0.08	0.75	3.12	1.46	4.15	28.29
Torquigener	0.96	0.29	2.94	1.4	3.91	32.2
Kyphosus	0	0.63	2.58	1.24	3.43	35.63
Acanthaluter	0.58	0	2.56	1.12	3.41	39.03
Plectorhinchu	0.17	0.63	2.48	1.14	3.3	42.33
Choerodon	0.08	0.54	2.31	1.02	3.07	45.41
Pomacentrus	0	0.54	2.28	1.02	3.04	48.45
Pseduocaran	0.29	0.54	2.26	0.99	3.01	51.46
Coris	0.54	1	2.22	0.88	2.95	54.41
Pentapodus	0.38	0.5	2.19	0.95	2.91	57.32

Sphyraena	0.5	0.04	2.17	0.96	2.88	60.2
Dasyatis	0.42	0.17	2.01	0.86	2.67	62.87
Seriola	0.17	0.38	1.8	0.82	2.4	65.27
Pelsartia	0.38	0.04	1.68	0.76	2.24	67.51
Neatypus	0	0.38	1.52	0.75	2.03	69.54
Siganus	0	0.38	1.46	0.76	1.94	71.48
Lethrinus	0.04	0.33	1.36	0.71	1.81	73.29
Myliobatis	0.25	0.08	1.25	0.62	1.67	74.95
Gymnothora:	0.04	0.29	1.23	0.66	1.64	76.59
Epinephelus	0.17	0.17	1.21	0.6	1.61	78.2
Ostorhinchus	0.29	0	1.17	0.63	1.56	79.77
Anampses	0	0.29	1.17	0.63	1.55	81.32
Pagrus	0.17	0.17	1.1	0.61	1.46	82.78
Abudefduf	0	0.25	1	0.57	1.34	84.12
Rhabdosargu	0.08	0.21	0.99	0.58	1.32	85.44
Arripis	0.21	0	0.88	0.5	1.17	86.61
Meuschenia	0.04	0.17	0.86	0.48	1.15	87.76
Lagocephalus	0.13	0.08	0.81	0.47	1.08	88.84
Chaetodon	0	0.17	0.79	0.43	1.05	89.89
Scorpis	0	0.17	0.71	0.44	0.94	90.83
